# Optimized Parallelization for Nonlocal Means Based Low Dose CT Image Processing

**DOI:** 10.1155/2015/790313

**Published:** 2015-05-19

**Authors:** Libo Zhang, Benqiang Yang, Zhikun Zhuang, Yining Hu, Yang Chen, Limin Luo, Huazhong Shu

**Affiliations:** ^1^Department of Radiology, General Hospital of Shenyang Military Area Command, Shenhe District, Shenyang 110840, China; ^2^Laboratory of Image Science and Technology, Southeast University, Nanjing 210096, China; ^3^The Key Laboratory of Computer Network and Information Integration, Southeast University and Ministry of Education, Nanjing 210096, China; ^4^Centre de Recherche en Information Biomedicale Sino-Francais (LIA CRIBs), 35000 Rennes, France

## Abstract

Low dose CT (LDCT) images are often significantly degraded by severely increased mottled noise/artifacts, which can lead to lowered diagnostic accuracy in clinic. The nonlocal means (NLM) filtering can effectively remove mottled noise/artifacts by utilizing large-scale patch similarity information in LDCT images. But the NLM filtering application in LDCT imaging also requires high computation cost because intensive patch similarity calculation within a large searching window is often required to be used to include enough structure-similarity information for noise/artifact suppression. To improve its clinical feasibility, in this study we further optimize the parallelization of NLM filtering by avoiding the repeated computation with the row-wise intensity calculation and the symmetry weight calculation. The shared memory with fast *I*/*O* speed is also used in row-wise intensity calculation for the proposed method. Quantitative experiment demonstrates that significant acceleration can be achieved with respect to the traditional straight pixel-wise parallelization.

## 1. Introduction

X-ray Computed Tomography (CT) can reflect human attenuation map in millimeter level, in which rich 3D information of tissues, organs or lesions can be provided for clinical diagnosis. Though its wide application in clinics, the radiation delivered to patients during CT examinations is always a wide-spread concern. It was reported in [1] that CT radiation may increase the risk of developing metabolic abnormalities even cancer. The most practical means to lower radiation dose is to decrease tube current (milliampere (mA)) or tube current time products (milliampere second (mAs)). However, lowering mA/mAs settings often leads to degraded CT images with increased mottled noise and streak artifacts [2, 3], which will influence the diagnosis accuracy [4–7]. Researchers can suppress noise and artifacts in low dose CT (LDCT) images by developing new reconstruction or postprocessing algorithms. Current solutions to improve the quality of LDCT images can be roughly divided into three categories: preprocessing approaches, iterative reconstruction approaches, and postprocessing approaches.

The first one refers to those techniques that improve CT imaging by suppressing the noise in projected raw data before the routine FBP reconstructions. The key of these techniques is to find the accurate statistical distribution of projected data and design effective restoration algorithms [5, 6]. The second one refers to iterative reconstruction approaches which treat the LDCT imaging as an ill-posed inverse problem and solve the problem as a prior-regularized cost function via some iterative optimization solutions [7, 8]. Though effective in obtaining favorable reconstructed image quality, the most well-known limit for iterative reconstructions is the required intensive computation in iterative optimization. Additionally, for patent protection consideration, current mainstream CT device suppliers normally do not provide well-formatted projected data, which severely restricts the research and the possible clinical application of these two study directions.

The third one refers to postprocessing methods, which can be directly applied to improve LDCT images. Distribution and scale features of noise, artifacts, and normal tissues in CT images need to be jointly considered in designing effective postprocessing algorithms [9, 10]. It was pointed in [9–14] that the nonlocal means (NLM) filtering, which utilizes the information redundancy property, can effectively suppress noise and artifacts without obviously blurring image details. We would also note that the patch similarity metric in NLM has also been used to build regularization term for tomographic reconstruction [15, 16].

However, since noise and artifacts often distribute with prominent amplitudes in LDCT images, a large searching window is practically required to include more structure information in noise/artifact suppression, which implies a large computation cost. This will strongly limit its clinical application considering the large workload in current radiology departments. To overcome this, this paper presents an improved GPU-based parallelization approach to accelerate the NLM filtering. The proposed approach optimizes the computation in NLM filtering by avoiding repeated computation with row-wise intensity calculation and weight calculation. The fast *I*/*O* data access speed for shared memory in GPU is also well exploited to reduce data *I*/*O* operation cost. Experiment results on 2D LDCT images demonstrate that the improved parallelization can significantly shorten computation time and, thus, making itself a potentially applicable processing procedure in LDCT imaging.

## 2. Nonlocal Means Based Low Dose CT Image Processing

Compared to restoration algorithms based on intensity gradient information, NLM filtering can provide edge-preserving noise/artifact suppression without blurring image structures. In NLM filtering, one image patch is matched with a group of similar patches in a large neighboring area, and in this way more structure similarity information in large neighboring scale can be used to suppress noise and artifacts in LDCT images. The NLM algorithm replaces pixel intensities by the weighted average of intensities within a searching window *N*. Each weight expresses the similarity between the central pixel and the neighboring pixels in the searching window and is calculated by the Euclidian distance between patches surrounding these two pixels. Let *p*  (*p* = (*p*_*x*_, *p*_*y*_)) denote the pixel to be processed, let *q* denote a pixel in the search neighborhood window, let *X* denote the processed image, and let *Y* denote the image to be processed; the 2D NLM filtering algorithm can be formulated as follows [17]:

(1) $$\begin{array}{c} \hat{X}\left(p\right)=\frac{\sum_{q\in N_{p}}\omega \left(p,q\right)Y\left(q\right)}{\sum_{q\in N_{p}}\omega \left(p,q\right)},\;\;\; \end{array}$$

(2) wp,q =exp⁡−∑Δx,Δy∈−B,…,B2dp,qΔx,ΔyGΔx,Δyh2B+12B+1,

(3) $$\begin{array}{c} d_{p,q}^{\left(\Delta x,\Delta y\right)}=Y\left(p_{x}+\Delta x,p_{y}+\Delta y\right)-Y\left(q_{x}+\Delta x,q_{y}+\Delta y\right), \end{array}$$

(4) $$\begin{array}{c} G\left(\Delta x,\Delta y\right)=\left\{\begin{array}{cc} \sqrt{2} & \left(\Delta x,\Delta y\right)=\left(0,0\right)\\ \frac{1}{\sqrt{\Delta x^{2}+\Delta y^{2}}} & \left(\Delta x,\Delta y\right)\neq \left(0,0\right), \end{array}\right.\;\;\; \end{array}$$

where *N*_*p*_ denotes the searching window centered at *p*; *w*(*p*, *q*) denotes the similarity of the two patches centered at *p* and *q*, respectively, with the radius *B*; *G*(Δ*x*, Δ*y*) is a distance dependent Gaussian kernel function; the number of pixels in a patch is (2*B* + 1)(2*B* + 1). We routinely use the parameter *h* in (2) to control the smoothing effect.

**Figure 1:**
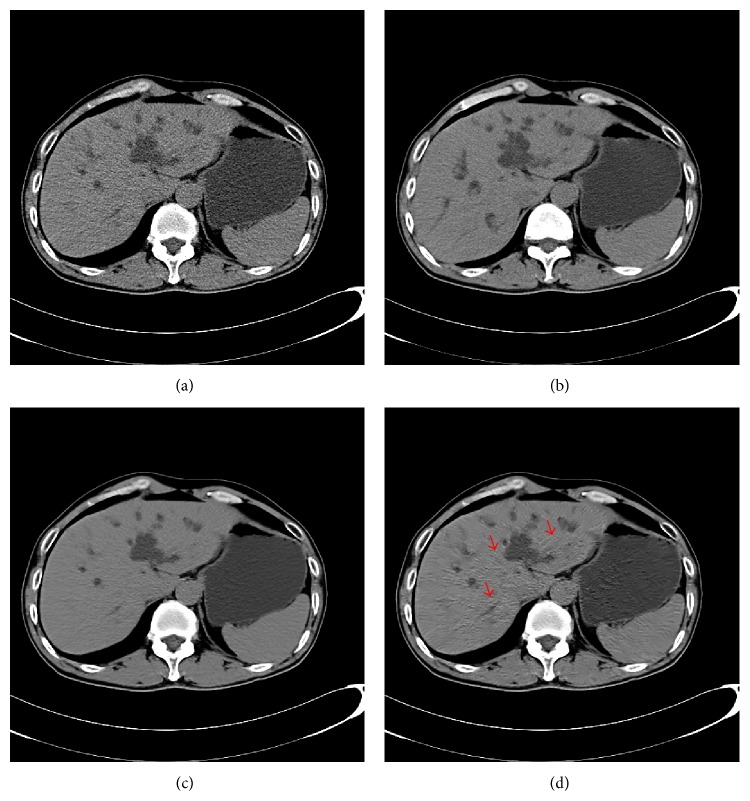
Processed result of one 512 × 512 abdomen LDCT image. (a) and (b) are the original LDCT image and the corresponding SDCT image. (c) and (d) are the LDCT images processed by NLM filtering with 81 × 81 and 81 × 81  21 × 21 searching windows, respectively.

**Figure 2:**
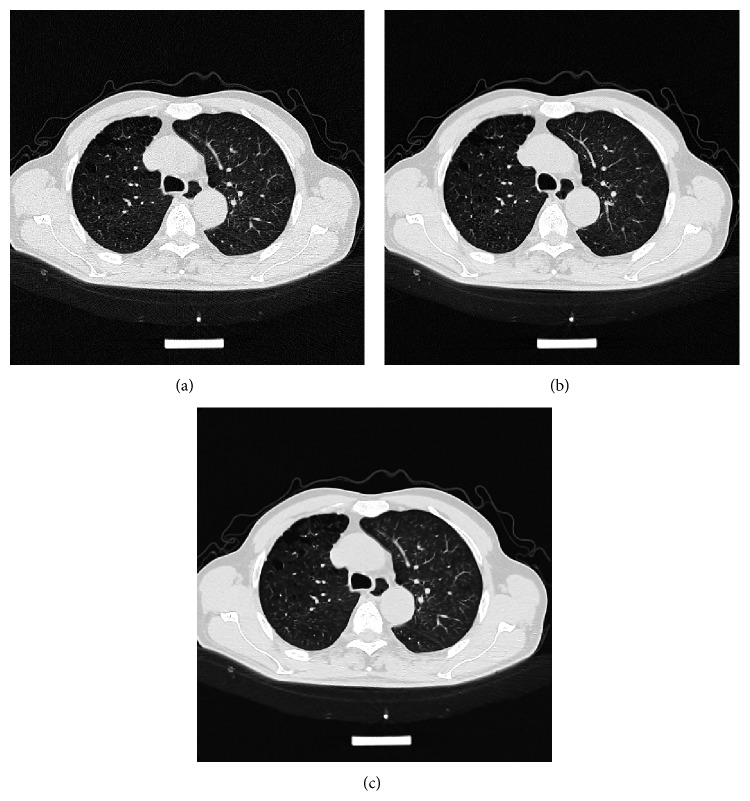
Processed result of one 512 × 512 thoracic LDCT image. (a), (b), and (c) correspond to the original LDCT image, the corresponding SDCT image, and the LDCT image processed by NLM filtering, respectively.

**Figure 3:**
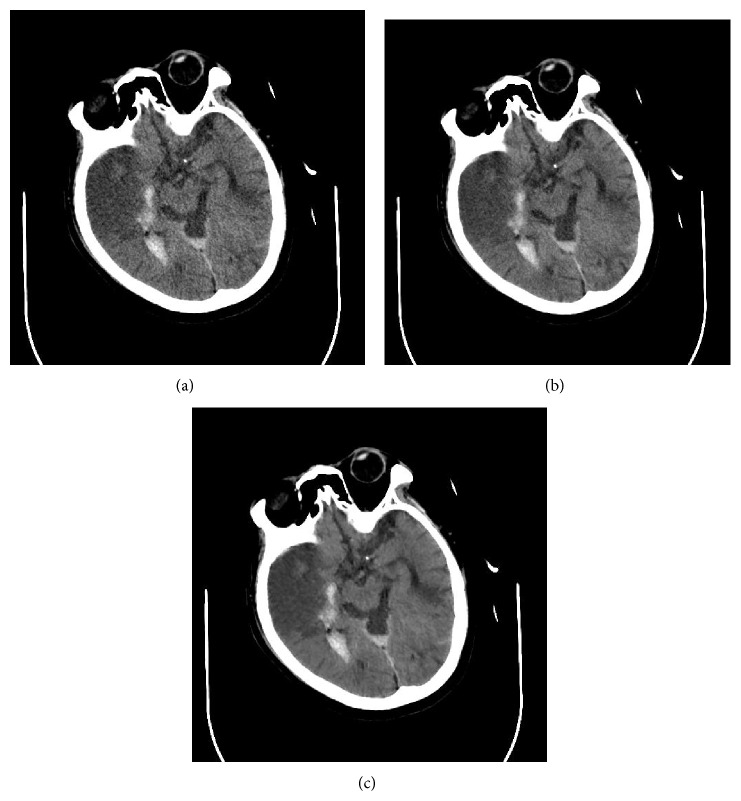
Processed result of one 512 × 512 brain LDCT image. (a), (b), and (c) correspond to the original LDCT image, the corresponding SDCT image, and the LDCT image processed by NLM filtering, respectively.

**Figure 4:**
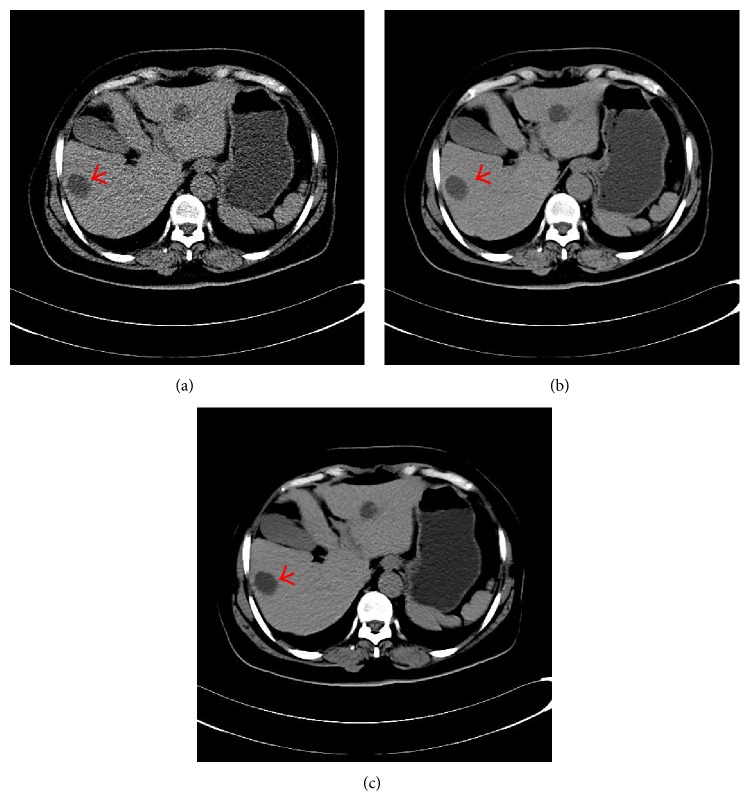
Processed result of one 512 × 512 abdomen LDCT image with tumor (pointed by arrows). (a), (b), and (c) correspond to the original LDCT image, the corresponding SDCT image, and the LDCT image processed by NLM filtering, respectively.

**Figure 5:**
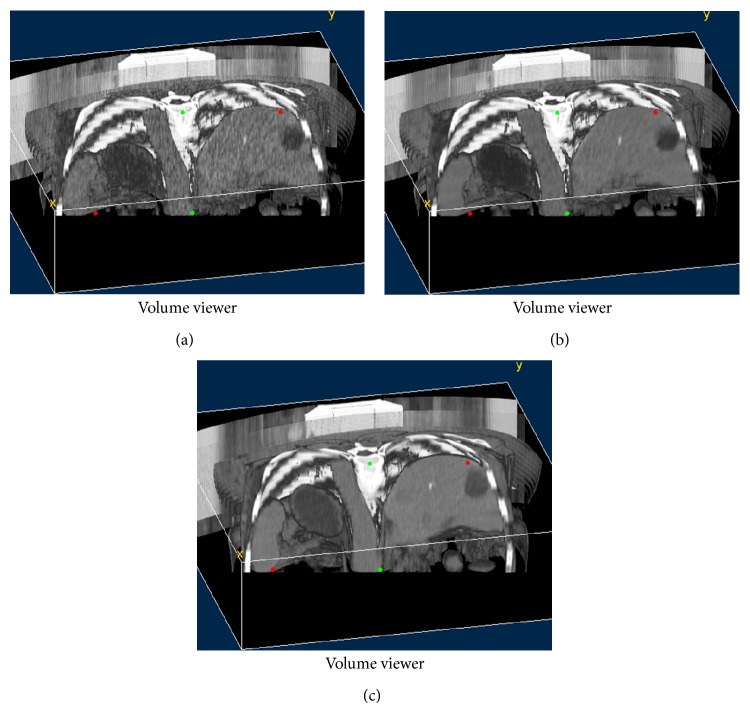
3D illustration of a set of thoracic LDCT images. (a), (b), and (c) correspond to the original LDCT volume, the corresponding SDCT volume, and the LDCT volume processed by NLM filtering, respectively.

In Figures 1–4, we give the NLM filtering processed results of four 2D LDCT images in Figures 1(a), 2(a), 3(a), and 4(a) and the two corresponding standard dose CT (SDCT) images are given in Figures 1(b), 2(b), 3(b), and 4(b) as references. The LDCT and SDCT images were collected using the reduced tube current 80 mA and the routine tube current 240 mA, respectively. We can see that CT images are mainly composed of pixels with limited intensity range, and the intensities representing different tissues spread over the whole image domain. Other scanning parameters were set by default. Compared to the reference SDCT images, we can see that tube current reduction will lead to severely increased noise and artifacts in LDCT images. Figures 1(c), 2(c), 3(c), and 4(c) illustrate the processing results of NLM filtering, in which the size of searching window is 81 × 81, patch size is 9 × 9 (*B* = 4), and parameter *h* is set to 10.  Figure 5 shows the results of 3D volumes by processing a set of 2D thoracic LDCT images. The illustrations in Figures 1–4 are presented in suitable windows. All the parameters were set under the guide of an experienced doctor in radiology department. We can see that the NLM filtering can effectively suppress both mottled noise and artifacts in LDCT images without leading to significantly blurred structures.

To highlight the importance of a large searching window, we also list in Figure 1(d) the NLM processed result with 21 × 21 searching window (other parameters are set to be same as the result in Figure 1(c)). We can see that processing with this smaller 21 × 21 searching window fails to give satisfying artifact suppression (see the arrows). Therefore, a large searching window (up to 81 × 81) needs to be used to in NLM filtering to include enough large-scale similarity information to suppress noise and artifacts in LDCT images, as can be seen in Figure 1(c) [10]. However, the patch similarity calculation within a large searching window is always accompanied with large computation load. For a *m* × *n* sized image, with the pixel number of searching window being |*N*| and patch radius being *B*, we get the computational complexity *O*(*mn*|*N*|(2*B* + 1)(2*B* + 1)) for the original CPU based serial processing, and the total computational complexity amounts to *O*(512 × 512 × 81 × 81 × 9 × 9) = *O*(1.3931 × 10^11^) for 512 × 512 sized images. This computation cost is too high to provide real-time CT imaging for radiology department routine; so we need to accelerate the NLM filtering in order to give fast clinical application.

## 3. CUDA-Based GPU Acceleration for NLM Algorithm

### 3.1. Introduction to CUDA-Based GPU Acceleration

Utilizing GPU based techniques to parallelize algorithm has already become a notable trend in the field of parallel computing. The GPU based parallelization is achieved by jointly parallelizing coarse-scale patches and fine-scale threads in the original grid computation task which is parallelizable [18–20]. The CUDA (Compute Unified Device Architecture) technology provides a software platform for developers to design parallelized tasks with C-style code, with direct access to the virtual instruction set and GPU memories. Each parallelization function running on CUDA is called a kernel, and we use *f* ^(*I*)^∘*f* ^(*I* − 1)^∘⋯∘*f* ^(1)^ to represent the connected parallelized cascade functions based on [20]. The output of kernel function *f* ^(*i*)^ is the input of *f* ^(*i*+1)^. We use (*U*_1_^(*i*)^,…, *U*_*k*_^(*i*)^) to represent the input data of kernel function *f* ^(*i*)^ in processing and (*U*_1_^(*i*+1)^,…, *U*_*k*_^(*i*+1)^) to represent the output data of function *f* ^(*i*)^. We can use (5) to represent the kernel function as follows:

(5) $$\begin{array}{c} \left[U_{1}^{\left(i+1\right)},\ldots ,U_{k}^{\left(i+1\right)}\right]\left(p\right)=f_{U_{1}^{\left(i\right)},\ldots ,U_{k}^{\left(i\right)}}^{\left(i\right)}\left(p\right),\; \end{array}$$

where *p* represents the pixel position in image. It should be noted that in some cases not all the input data need to be updated (e.g., *U*_*j*_^(*i*+1)^ = *U*_*j*_^(*i*)^).

### 3.2. Conventional GPU Based Parallelization for NLM Filtering Algorithm

The conventional GPU based parallelization accelerates the NLM filtering algorithm by direct pixel-wise parallelization. Based on above (1)–(4), we routinely break the algorithm into four parts specified by the following (6)–(9), which are computed in loops. The number of loops is set as the searching window size |*N*| to traverse all the neighboring points in window *N*. The first kernel function (6) computes intensity differences in parallel via GPU and has computational complexity *O*(1). Here we quantify the computational complexity using the operation times in parallel. In (6), (*p*_*x*_ + *i*_*x*_, *p*_*y*_ + *i*_*y*_) denotes the spatial position of the neighboring pixel in the searching window centered at *p*, which can also be represented by spatial position (*p*_*x*_, *p*_*y*_). We set *U*_3_^(1)^ = *U*_4_^(1)^ = 0 as initialization. Consider

(6) U13i−1,…,U43i−1p  =fU13i−2,…,U43i−23i−2p  =Ypx,py−Ypx+ix,py+iyU23i−2pU33i−2pU43i−2p.

For data *U*_2_, the second kernel function computes the patch similarity using (7) based on the patch difference computed by (6) in *U*_1_. We can see that the computational complexity of the second kernel function is around *O*((2*B* + 1)(2*B* + 1)) because there are (2*B* + 1)(2*B* + 1) weighted summation operations for each pixel pair in the two comparing patches. Consider

(7) U13i,…,U43ip=fU13i−1,…,U43i−13i−1p=3i−1exp⁡−∑(Δx,Δy)∈[−B,…,B]2U13i−1px+Δx,py+ΔyGΔx,Δyh2B+12B+1U33i−1U43i−1.

The third kernel function (8) computes the summation of weights and intensities in *U*_3_ and *U*_4_, and the computational complexity is *O*(1) for this operation. Consider

(8) U13i+1−2,…,U43i+1−2p  =fU13i,…,U43i3ip  =U13ipU23ipU33ip+U23ipU43ip+U23ipYpx+ix,py+iy.

In the final loop *I* = |*N*| + 1, a last kernel function in (9) is applied to compute the final output image $\hat{X}(p).\;$ Consider

(9) $$\begin{array}{c} f_{U_{1}^{\left(I\right)},\ldots ,U_{4}^{\left(I\right)}}^{\left(I\right)}\left(p\right)=\left(\begin{array}{c} 0\\ 0\\ 0\\ \frac{U_{4}^{I}\left(p\right)}{U_{3}^{I}\left(p\right)} \end{array}\right). \end{array}$$

Here *I* represents the last loop number. The computational complexity for the operation (9) is also *O*(1). The final image is outputted as $\hat{X}(p)=U_{4}^{\left(I\right)}(p)/U_{3}^{\left(I\right)}(p)$. Combing all the operations from (6)–(9), we can see that the whole computational complexity of the conventional parallelization algorithm amounts to *O*(|*N*|((2*B* + 1)(2*B* + 1) + 2) + 1).

### 3.3. Improved GPU Acceleration for NLM Filtering Algorithm

**Figure 6:**
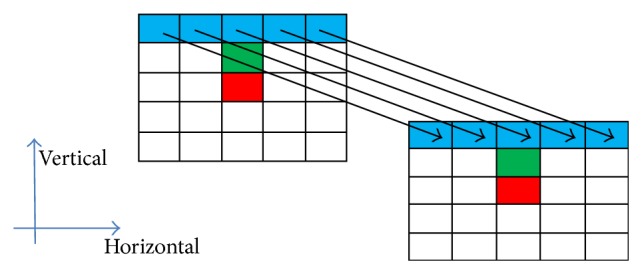
Row-wise calculation in patch difference calculation.

In the above conventional parallelization approach, the second kernel function in (7) is serially applied to compute the patch similarity, which leads to large computation cost when large searching window is used. Our first improvement is, thus, devoted to reduce the computational complexity in this part.  Figure 6 illustrates that a patch is of size 5 × 5 (*B* = 2) with the red point indicating the center point. Equations (1)–(4) show that the patch similarity in NLM filtering can be quantified by the weighted sum of intensity differences of the corresponding pixels in the two patches. In Figure 6, we can see that, for the center points located at the green points in the two patches, the summed intensity difference of the blue points in the same rows is in fact the same value as the case when the center points moves down to the red points. This implies that the intensity differences of rows are repeatedly computed when the center points move within (*B* + 1) pixel distances. Therefore, we can efficiently calculate patch differences via the following row-wise calculation:

(10) ⋃Δy∈0,…,B ∑Δx∈−B,…,BYpx+Δx,py−Yqx+Δx,qywwwwwwwwww×GΔx,Δy,

where the intensity difference between two individual pixels is |*Y*(*p*_*x*_ + Δ*x*, *p*_*y*_) − *Y*(*q*_*x*_ + Δ*x*, *q*_*y*_)| and *q* = (*q*_*x*_, *q*_*y*_) represents the neighboring point positions in the searching window. ⋃_Δ*y*∈[0,…,*B*]_  denotes the dataset that includes the (*B* + 1) different points in the vertical direction. Thus, with patches of size (2*B* + 1) × (2*B* + 1), we know from (10) that (*B* + 1) values can be obtained for (*B* + 1) different row pairs.* The row difference needs to be calculated only once before being stored in the shared memory, and the other B operations in (10) can be easily obtained by loading data from the shared memory and then performing Gaussian weighting*. For GPU with fast single-precision floating processing, the main computation cost of (10) lies in the data accessing operation of the global memory because the time cost in shared memory accessing is trivial when compared to global memory accessing. The computational complexity of (11) can be roughly estimated to be *O*(2*B* + 1) [21].

Similar to the conventional GPU parallelization, we also divide the algorithm into the following four parts (11)–(14) and compute in loops. Suppose that the input image is of size *m* × *n*, we set the size of *U*_1_^(*i*)^ to be *m* × *n* × (*B* + 1). The data *U*_2_^(*i*)^, *U*_3_^(*i*)^, *U*_4_^(*i*)^, *U*_5_^(*i*)^ are of size *m* × *n*, and (*p*_*x*_ + *i*_*x*_, *p*_*y*_ + *i*_*y*_) denotes the neighboring points in the searching window centered at *p*. The initialization is also set with data *U*_3_^(1)^ = *U*_4_^(1)^ = 0, *U*_5_^(*i*)^ = *Y*.

The first kernel function computes the sum of the intensity differences for each row pair, which is multiplied by the Gaussian weight calculated based on the perpendicular distance from the row to the center point. The computational complexity of this kernel function is *O*(2*B* + 1). Consider

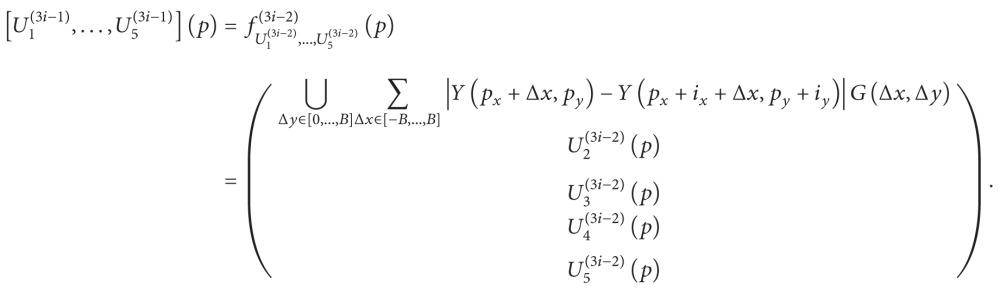
(11)


The second kernel function calculates the similarity of patches based on (12). In (12), we compute the patch similarity by accumulating the absolute values of the weighted sum of the intensity differences calculated via the first kernel function (11). The computational complexity of the kernel function (12) is *O*(2*B* + 1). Consider

(12) U13i,…,U53ip  =fU13i−1,…,U53i−13i−1p  =U13i−1pexp⁡−∑Δy∈−B,…,BU13i−1px,py,Δyh2B+12B+1U33i−1pU43i−1pU53i−1p.

The second improvement is saving one half computation cost by exploiting the symmetry property of weights calculated in (2). Apparently, we have *w*(*p*, *p* + Δ*p*) = *w*(*p* + Δ*q*, *p*) (Δ*q* represents the location offset of pixel *p* in the searching window). Based on this symmetry property *w*(*p* − Δ*q*, *p*)*Y*(*p* − Δ*q*) = *w*(*p*, *p* − Δ*q*)*Y*(*p* − Δ*q*), we also accumulate *w*(*p* − Δ*q*, *p*)*Y*(*p* − Δ*q*) when accumulating weighted intensity *w*(*p*, *p* + Δ*q*)*Y*(*p* + Δ*q*) for location *p*. In this way, we only need to go through half of the pixels in the searching window. The third kernel function is given by (13), whose computational complexity is *O*(1). Consider

(13) U13i+1−2,…,U53i+1−2p=fU13i,…,U53i3ip=U13ipU23ipU33ip+U23ip+Δq+U23ip−ΔqU43ip+U23ip+ΔqU53ip+Δq+U23ip−ΔqU53ip−ΔqU53ip.

Then, a final kernel function (14) can be applied to obtain the finally processed image:

(14) $$\begin{array}{c} f_{U_{1}^{\left(I\right)},\ldots ,U_{5}^{\left(I\right)}}^{\left(I\right)}\left(p\right)=\left(\begin{array}{c} 0\\ 0\\ 0\\ 0\\ \frac{U_{4}^{\left(I\right)}\left(p\right)}{U_{3}^{\left(I\right)}\left(p\right)} \end{array}\right). \end{array}$$

The final loop number with respect to the required operation number as to the searching window now becomes (2*T* + 1) × (*T* + 1) + 1 (*T* denotes the radius of the searching window), which is approximately 0.5|*N*|. The final output image is $\hat{X}(p)=U_{4}^{\left(I\right)}(p)/U_{3}^{\left(I\right)}(p)$. To conclude, the total computational complexity of the improved algorithm is around *O*(0.5|*N*|(2(2*B* + 1) + 1) + 1), which approximately equals to *O*(|*N*|(2*B* + 1) + 1). We can see that the computational complexity has been reduced to 1/(2*B* + 1) with respect to the conventional parallelization.

## 4. Experimental Results and Analyses

**Figure 7:**
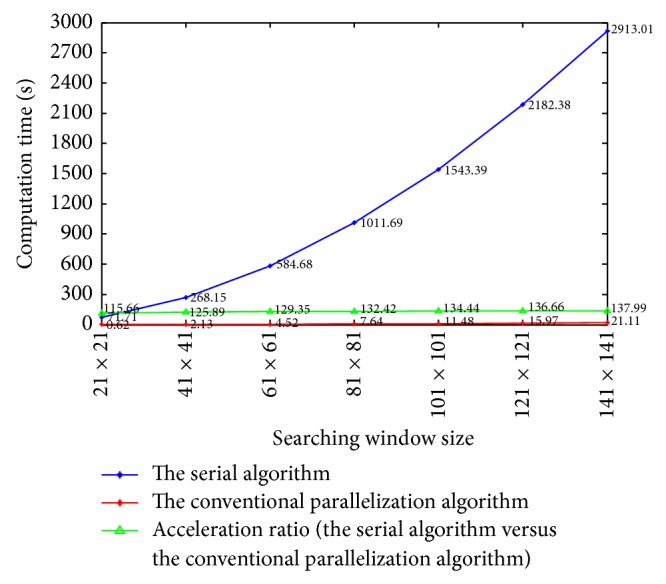
Comparison of the computation time of the serial algorithm and the conventional parallelization algorithm for NLM filtering.

In this section we compare the computation cost of different methods. To verify the improvement brought by the proposed acceleration method for the NLM filtering, we process the same 512 × 512 LDCT image in Figure 1(a) using the serial algorithm (CPU based), the conventional parallelization algorithm (GPU based), and the improved parallelization algorithm (GPU based). In this section, we do not illustrate the processed images because the same images as Figure 1(a) were obtained. We set the patch size to be 9 × 9 and record the computation time with respect to the size of searching window.  Figure 7 illustrates the computation time of the serial algorithm and the conventional parallelization algorithm. We can observe that the conventional parallelization significantly reduces the computation cost through straight pixel-wise parallelization and achieves an acceleration ratio of more than 100 times of the original serial algorithm. The system configuration for our experiments is given as follows.

### 4.1. Hardware Environment

CPU: Inter(R) Core(TM) i7-3770 CPU @ 3.40 GHz; Memory: 8 GB; Graphics Card: NVIDIA GeForce GTX 680 with 1536 CUDA cores; Effective memory clock: 6008 MHz; Memory bandwidth: 192 GB/s; Memory size: 2 GB; Memory bus type: 256 bit.

### 4.2. Software Environment

Operating System: Win7 64 bit; Matlab: R2011a; CUDA: 4.0.

**Figure 8:**
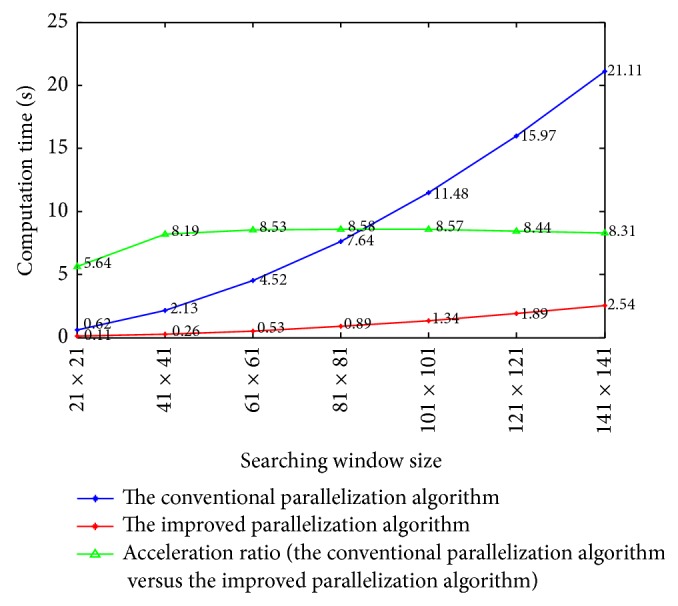
Comparison of the computation time with respect to searching window size for the conventional parallelization algorithm and the improved parallelization algorithm.

**Figure 9:**
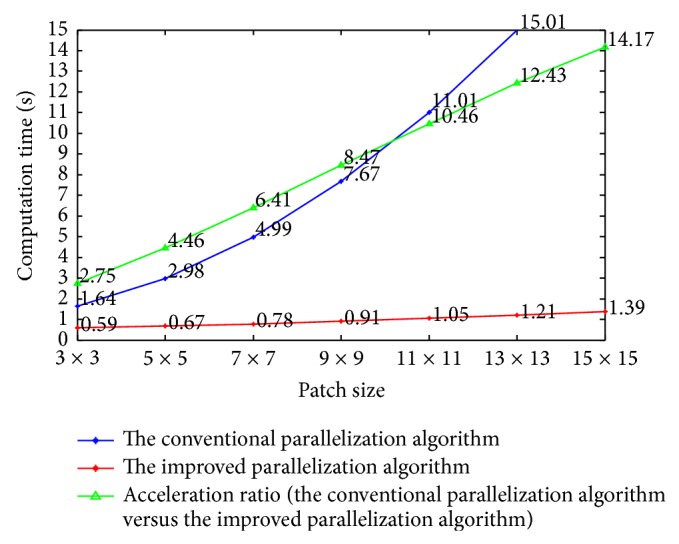
Comparison of the computation time with respect to patch size for the conventional parallelization algorithm and the improved parallelization algorithm.

Then, we compare the computation time of the conventional parallelization algorithm and the improved parallelization algorithm with respect to the size of searching window. The patch size is fixed to 9 × 9. As can be seen in Figure 8, when the searching window size becomes larger than 41 × 41, the acceleration ratio approximately equals to 2*B* + 1 = 2 × 4 + 1 = 9, and this observation is consistent with the above deduced acceleration ratio 2*B* + 1. In addition, we compare the computation time of the conventional parallelization algorithm and the improved parallelization algorithm with respect to patch size. The searching window size is fixed to 81 × 81. Since a large patch size often leads to blurred images, we hereby set the maximal patch size to be 15 × 15. We can observe in Figure 9 an obvious increment of acceleration ratio when the patch size increases, and this again verifies the above deduced acceleration ratio 2*B* + 1.

## 5. Discussion and Conclusion

In this paper we further optimize the parallelization for NLM filtering in CT image processing. The proposed approach optimizes the parallelized computation in NLM filtering by avoiding repeated computation with row-wise intensity calculation and weight calculation. The fast *I*/*O* data access speed for shared memory in GPU is also well exploited. We applied our improved algorithm to LDCT image processing and find that the improved algorithm can achieve a significant acceleration ratio with respect to the conventional parallelization algorithm. For now, it takes about 0.8 second to process one 512 × 512 CT image with 81 × 81 searching window and 9 × 9 patch, and this parameter setting in NLM filtering is found to be able to provide effective processing. This paper only provides the results on 2D NLM filtering, and we would stress that the same parallelization strategy can be easily extended to accelerate the more computationally intensive 3D NLM filtering, and the same acceleration ratio as 2D case can be expected because they have the same calculation structures. To be specific, this extension can be realized by replacing the row-wise optimization in (11) by a plane-wise optimization. Nevertheless, we would also point it out that 3D NLM filtering should not be suggested for the processing of CT slices with large slice thickness (>2 mm) because of the poor interslice continuity in this case.

Currently, the structure similarity idea in NLM has got wide applications in the other field of image processing (e.g., image segmentation and image reconstruction) [15, 16, 22, 23]. The proposed parallelization optimization can be directly applied to accelerate the patch similarity calculation in these applications. In current parallelization approach, the weights reflecting patch similarity are calculated via a serial loop in (7), which can be further parallelized via interkernel operations to realize a further acceleration. Accelerating the computation speed by combining multiple-core CPU strategy with GPU parallelization technique will also be explored. This optimization strategy can be easily used to accelerate other reconstruction or restoration tasks using the patch similarity type metrics [24–26]. We can also consider improving the performance of the NLM filtering by incorporating the fractional metric into the calculation of patch similarity [27]. Evaluation of the potential accuracy enhancement in segmentation/registration (related with CT images) that can be brought by the proposed processing also needs to be performed [28–30]. All these issues will be addressed in the future work.
